# H9N2 Viruses Isolated From Mammals Replicated in Mice at Higher Levels Than Avian-Origin Viruses

**DOI:** 10.3389/fmicb.2019.00416

**Published:** 2019-03-11

**Authors:** Haiwei Sun, Kai Wang, Wei Yao, Qinfang Liu, Jianmei Yang, Qiaoyang Teng, Xuesong Li, Zejun Li, Hongjun Chen

**Affiliations:** ^1^Shanghai Veterinary Research Institute, Chinese Academy of Agricultural Sciences, Shanghai, China; ^2^Animal Influenza Virus Ecology and Pathogenesis Innovation Team, The Agricultural Science and Technology Innovation Program, Shanghai, China

**Keywords:** influenza A virus, H9N2, subtype, genotype, pathogenicity

## Abstract

H9N2 subtype influenza A virus (IAV) has more than 20 genotypes that are able to cross species barriers and expand from birds to mammals and humans. To better understand the impact of different H9N2 genotypes and their characteristics, five H9N2 viruses from different hosts including chickens, geese, pigs, mink, and humans representing the B69 88(Gs/14, Ck/15, and Mi/14), B35 (Sw/08) and G9 genotypes (Hu/04) were infected in chicken and mice. In mice, mammal-origin viruses replicated at higher levels in the lungs compared to avian viruses. The goose-virus replicated at the lowest levels indicating poor adaptation. Increased pro-inflammatory cytokines were positively correlated with viral loads in the lung. In chickens, all viruses were excreted from cloacal and/or oropharyngeal swabs. Interestingly, Mink-origin virus exhibited higher virulence and replication in mice and chickens. Our data indicate that mammal-origin H9N2 viruses are more adapted and virulent in mice than the avian-origin viruses.

## Introduction

H9N2 was considered to be a low pathogenicity avian influenza virus (AIV) because it causes mild clinical disease (Cauthen et al., 2000). Poultry infected with AIV H9N2 are important incubators for novel emerging pandemic AIVs (Liu et al., 2014; Sun and Liu, 2015), which contribute to the reassortment of new human H7N9 or H10N8 influenza viruses (Pu et al., 2015). In mainland China, H9N2 virus was first isolated from chickens in Guangdong Province in 1994 (Zhao et al., 2015), and then from humans 5 years later in Hong Kong (Guo et al., 1999). H9N2 viruses reassort with a variety of other subtypes to form novel influenza virus genotypes. Moreover, H9N2 viruses were reported to have evolutionary human-like receptor identity with a Q226L substitution in the hemagglutinin (HA) glycoprotein and have adapted to bind mammalian host receptors, which led to an increase in human susceptibility (Matrosovich et al., 2001). Thus, the subtype was suggested to be a considerable threat to public health (Lin et al., 2000; Saito et al., 2001; Butt et al., 2005).

H9N2 influenza viruses have evolved by reassortment and mutation over 25 years of prevalence in poultry. The “fittest” genotype, G57, changed antigenicity, which resulted in viral escape from immunization and caused widespread outbreaks in 2010–2015 and finally became the single predominant genotype in Chinese farmed chickens. Moreover, the genotype G57 of H9N2 subtype AIV contributed its six internal segments to the novel H7N9 viruses by reassortment in 2013. The widespread use of vaccines against H9N2 viruses in chickens has exerted continuous immune pressure on the hemagglutinin (HA) glycoprotein with antigenic drift (Pu et al., 2015).

To date, little information has been available on the host response and cytokine production in response to AIV H9N2 infection. In this paper, the pathogenicity of H9N2 viruses was investigated in mice and chickens to determine immunological patterns with different genotypes.

## Materials and Methods

### Ethics Statement for Animal Usage

All animal studies were carried out in strict accordance with the recommendations in the guide of Shanghai Veterinary Research Institute, CAAS (ID: SHVRI-PO-2014-0098) and approved by the Animal Association of Science and Technology Commission of Shanghai Municipality, China (Permit Number: 2013-11).

### Cell Culture and Reagents

Human embryonic kidney 293T (HEK 293T) cell line and Madin Darby canine kidney (MDCK) cell line were maintained in Dulbecco’s modified Eagle’s medium (DMEM; HyClone, United States) containing 5% fetal bovine serum (FBS; PAN Biotech, Germany) and 1% penicillin-streptomycin solution (PS; Thermo Fisher Scientific., United States) at 37°C with 5% CO_2_.

### Viruses

Five H9N2 influenza A virus strains were isolated from different hosts. Strain Sw/08 was isolated from a Jiangsu swine farm in 2008 with mild respiratory disease and named A/swine/Jiangsu/Sw/08/08 (abbr. Sw/08). Strain Ck/15 was isolated from an Anhui chicken farm in 2015 and named A/chicken/Anhui/120/2015 (abbr. Ck/15). Strain Gs/14 was isolated from a Jiangsu goose farm in 2014 and named A/goose/Jiangsu/4539/2014 (abbr. Gs/14). Strain Hu/04 was isolated from humans in 2004 and named A/Guangdong/Hu/04/2004 abbr. Hu/04). Strain Mi/14 was isolated from mink in 2014 and named A/mink/China/01/2014 (abbr. Mi/14). H9N2 viruses were grown by inoculation in 9 days-old specific pathogen-free chicken eggs (Merialvital, Co., Beijing). The allantoic fluids were collected 48 h later and stored at −80°C until use (Jin et al., 2018). The viral titers were determined and calculated according to the Reed–Muench method (Reed and Muench, 1938; Brown, 1964) for a 50% tissue culture infectious dose (TCID_50_) on MDCK cells with MEM (Hyclone Laboratories, San Angelo, TX, United States) with 1 μg/ml trypsin treated with L-(tosylamido-2-phenyl) ethyl-chloromethyl ketone (TPCK) (Chen et al., 2014a).

### Sequencing and Phylogenic Analysis of the H9N2 Viruses

Total RNA was extracted by using the RNeasy kit (Qiagen, Inc., Gaithersburg, MD, United States) following the manufacturer’s instructions. Reverse transcription was carried out using the Uni12 primer (5′-AGCAAAAGCAAGG-3′) and avian myeloblastosis virus (AMV) reverse transcriptase (Takara) following the manufacturer’s instructions and the cDNA products were stored at −80°C until use. The eight segments of influenza A virus were amplified by PCR using universal primers (Wang et al., 2017) and Phusion high-fidelity DNA polymerase (Vazyme, Inc., Nanjing, China). The PCR products were cloned into the pHW2000 vector using an *in vitro* recombination approach (Wang et al., 2017). The cloned plasmids were confirmed by sequencing with the Sanger method (Genewiz, Inc., Suzhou, China) with primers: 5′-CGCAAATGGGCGGTAGGCGTG-3′ (CMV-Forward) and 5′-TAGAAGGCACAGTCGAGG-3′ (BGH-Reverse). The eight segments of H9N2 virus were analyzed and aligned by using MEGA6 software (Tamura et al., 2013).

### Pathogenicity of H9N2 Viruses in Chickens

To identify the pathogenicity of H9N2 viruses in chickens, Eight SPF chickens (4-week-old) per group were intranasally inoculated at an equal dose of 1 × 10^6^ TCID_50_ per 100 μl diluted in PBS buffer. Negative control chickens received 100 μl PBS. After infection, SPF chickens were monitored daily for 14 days. Oropharyngeal and cloacal swabs were collected at 1, 3, 5 days post-inoculation (dpi) and then stored at −80°C in 1 ml PBS buffer containing 100 U/ml penicillin and 0.05 mg/ml streptomycin. Three chickens from each challenge group were randomly euthanized at 3 dpi and the trachea samples were collected and fixed in 10% formalin and subsequently embedded in paraffin for hematoxylin and eosin (H&E) staining. Meanwhile, the lung samples were collected and homogenized using a Tissue Lyser at 70 times 1/s for 2 min (Chen et al., 2014a), and centrifuged at 13, 200 × *g* for 10 min, 100 μl aliquots of the supernatants were collected and the titers were determined on MDCK cells. The pathogenic scores were measured based on the distribution of tracheal lesions (Gibson-Corley et al., 2013). The remaining chickens were monitored daily for clinical signs then euthanized at 14 dpi.

The infected lung tissues were homogenized and total RNA was extracted (Sun et al., 2017). The cDNA synthesis was driven by AMV transcriptase (Takara) with Oligo(dT)_18_ in 20 μl volumes at 30°C for 10 min, 42°C for 2 h, and then 70°C for 15 min. The primers of qRT-PCR were designed using PrimerQuest Tool. The sequences of the primers were as follows: qckβ-actin-F: TCCCTGGAGAAGAGCTATGAA; qckβ-actin-R: CAGGACTCCATACCCAAGA AAG. qckIL-1β-F: CTCACAGTCCTTCGACATCTTC; qckIL-1β-R: TGTTGAGCC TCACTTTCTGG. qckIL-6-F: TCTGTTCGCCTTTCAGACCTA; qckIL-6-R: GACCACCTCATCGGGATTTAT. qckTNF-α-F: CCGCCCAGTTCAGATGAGTT; qckTNF-α-R: GCAACAACCAGCTATGCACC. qckMCP-1α-F: CCCACAACTGCC ATAGATGAA; qckMCP-1α-R: GCTTTCCCGAGGGCAATAA; qckMIP-1-F: GGGCTCTGTTTCTCTCTCTTC; qckMIP-1-R: CATTTGCTGCTGGTGATGTAG. The reactions were conducted by qRT-PCR analysis with AceQ^®^ qPCR SYBR^®^ Green Master Mix (Vazyme) according to the following cycle protocol: 95°C for 5 min, 40 cycles at 95°C for 10 s, and 60°C for 30 s or followed by the melt curve stage (95°C for 15 s, 60°C for 1 min, and 95°C for 15 s). The reaction results were represented by threshold cycle (Ct) values. The fold change was calculated using the 2^−ΔΔCt^ method (Huang et al., 2017). Mean Ct values were determined based on triplicates.

### Flow Cytometry

Chicken peripheral blood mononuclear leukocytes (PBML) were isolated from chicken wing veins using a Ficoll-paque (Amersham Pharmacia Biotech, Piscataway, NJ, United States) with a density gradient of 50 × g at 4°C for 30 min, and washed three times with PBS containing 1% FBS. PBML cells were incubated with FITC-labeled anti-chicken CD3 antibody (1:50) (Southern Biotech, Cambridge, United Kingdom), PE-labeled-anti chicken CD4 antibody (1:50) (Southern Biotech), and APC-labeled-anti chicken CD8 antibody (1:50) (Southern Biotech) for 20 min at 4°C, washed with PBA (PBS, 0.5% BSA, and 0.0001% sodium azide) and then incubated with a goat anti-chicken IgG (Southern Biotech) for 20 min at 4°C. The stained cells were washed with PBS buffer and fixed with 2% paraformaldehyde (Merck, Inc., Germany) for 10 min at room temperature. The cells were washed once in PBA buffer. At least 10,000 events were acquired using a FACS Calibur flow cytometer (BD Biosciences, San Jose, CA, United States) and the data were analyzed using FlowJO (Three Star, Inc., Ashland, OR, United States).

### Pathogenicity of H9N2 Viruses in Mice

To determine the virulence of the H9N2 viruses *in vivo*, eight 5-week-old female BALB/c mice (Vital-River, Co., Beijing) were infected intranasally. BALB/c mice were anesthetized with dry ice prior to intranasal inoculation (Jones et al., 1999; Kohler et al., 1999), each received 1 × 10^6^ TCID_50_ per 50 μl intranasally (13 mice/group), with 50 μl PBS given to negative control mice. To determine virus titers from the lung, three mice per group were sacrificed at 3 dpi (Chen et al., 2014a). The lung samples were collected and homogenized using a Tissue Lyser at 70 times 1/s for 2 min (Chen et al., 2014a), and centrifuged at 13, 200 × *g* for 10 min, 100 μl aliquots of the supernatants were collected. The body weights of animals were monitored daily until 14 days after inoculation, and animals with more than 25% body weight loss were euthanized. The viral titers were titrated on MDCK cells. The levels of IL-1β, IL-6, MIP-1α, MCP-1, and TNF-α in the 100 μl aliquots of the homogenized lung samples were measured using sandwich enzyme-linked immunosorbent assay (sELISA) kits (eBioscience, Inc., San Diego, CA, United States) according to the manufacturer’s recommendations.

### Statistical Analysis

Statistical analyses were performed using Prism 6 software (GraphPad, La Jolla, CA, United States). Pairwise comparisons between groups means were carried out with a two-stage step-up procedure by Benjamini test, whereas multiple comparisons were carried out by an analysis of variance (one-way ANOVA method). The differences were considered significant at *p*-values of < 0.05 or < 0.01.

## Results

### Phylogenetic Analysis of H9N2 Subtype IAV Genotypes

To further characterize the different genotypes of H9N2 subtype influenza A viruses, eight segments of each selected virus were amplified, sub-cloned, and sequenced. The sequences were deposited into GenBank with accessions of the isolate KK (KX867822–KX867829), the isolate Ck/15 (KX867830–KX867837), the isolate Gs/14 (KX867838–KX867845), and the isolate Hu/04 (KX867846–KX867853). The characterization of Mi/14 virus (MF996796–MF996803) was confirmed in a recent report (Xue et al., 2018).

**FIGURE 1:**
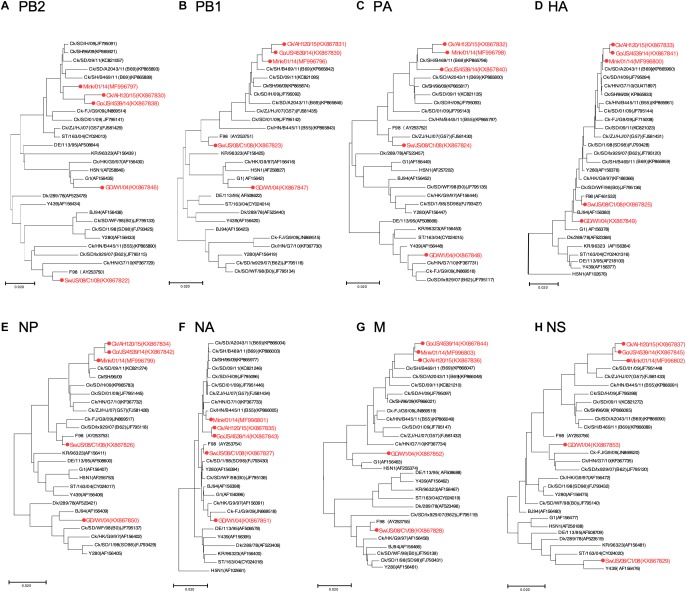
Phylogenetic analysis of H9N2 influenza viruses. Phylogenetic analysis of all the eight segments was constructed using the maximum Likelihood method by MEGA6.0 with 1,000 bootstrap replicates. **(A–H)** Phylogenetic trees for genes PB2, PB1, PA, HA, NP, NA, M, and NS, with analysis based on the segment of the corresponding gene given in nucleotides. The reference sequences used for genetic comparison were obtained from GenBank. AH, Anhui; Ck, Chicken; Dk, Duck; Goose, Gs; Human, Hu; FJ, Fujian; GD, Guangdong; HN, Hunan; HK, HongKong; JS, Jiangsu; Qa, Quail; SH, Shanghai; ST, Shantou; Sw, Swine; ZJ, Zhejiang; 03, 2003; 04, 2004; 06, 2006; 09, 2009; 11, 2011; 12, 2012; 13, 2013; 14, 2014; 15, 2015.

**FIGURE 2:**
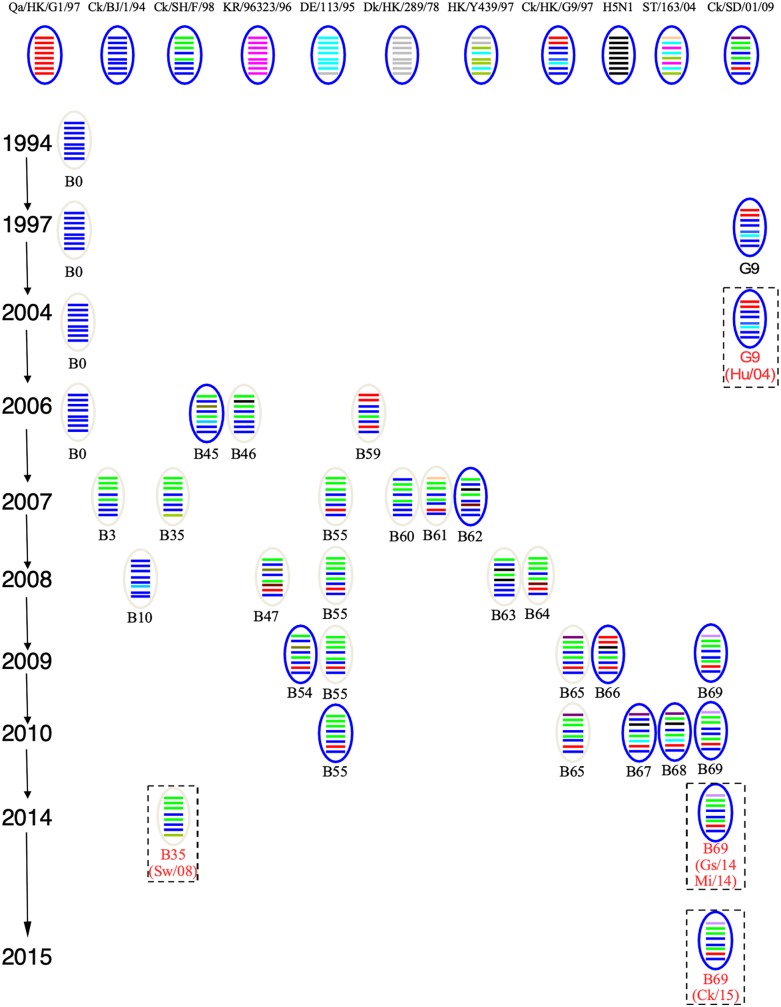
Genotypes of H9N2 influenza viruses. In the ovals, horizontal bars from top to bottom stand for PB2, PB1, PA, HA, NP, NA, M, and NS. A/Quail/Hong Kong/G1/1997 (Qa/HK/G1/97) was isolated from quail in 1997 in Hong Kong, the NCBI taxon ID was 97352 and representing genotype G1. A/Chicken/Beijing/1/1994 (Ck/BJ/94) was isolate from chicken in 1994 in Beijing, the NCBI taxon ID was 1342391 and representing genotype BJ94. A/Chicken/ Hong Kong/G9/1997 (Ck/HK/G9/97) was isolate from chicken in 1997 in Hong Kong, the NCBI taxon ID was 1342394 and representing genotype G9. A/Chicken/Korea/38349-p96323/1996 (Ck/KR/96323/96) was isolate from chicken in 1996 in Korea, the NCBI taxon ID was 97358 and representing genotype C0. A/Chicken/Shandong/01/2009 (Ck/SD/01/09) was isolate from chicken in 2009 in Shandong, the NCBI taxon ID was 1005638 and representing genotype B65. A/Chicken/Shanghai/F/1998 (Ck/SH/F/98) was isolate from chicken in 1998 in Shanghai, the NCBI taxon ID was 183741 and representing genotype F/98. A/Chicken/Zhejiang/B2013/2012 (Ck/ZJ/B2013/12) was isolate from chicken in 2013 in Zhejiang, the NCBI taxon ID was 1621010 and representing genotype B69. A/Duck/Hong Kong/289/1978 (Dk/HK/289/78) was isolate from duck in 1978 in Hong Kong, the NCBI taxon ID was 210667 and representing genotype E0. A/Duck/ Hong Kong/Y439/1997 (Dk/HK/Y439/97) was isolate from duck in 1997 in Hong Kong, the NCBI taxon ID was 1342412 and representing genotype Y439. A/Duck/Germany/113/1995 (Dk/GE/113/95) was isolate from duck in 1978 in Germany, the NCBI taxon ID was 1175984 and representing genotype D1. A/Duck/ Fujian/ C2246 (DK/FJ/C2246/09) was isolate from duck in 2009 in Fujian, the NCBI taxon ID was 1521103 and representing genotype B67. A/Duck/Shantou/163/2004 (Dk/ST/163/04) was isolate from duck in 2004 in Shantou, the NCBI taxon ID was 456814 and representing genotype ST/163. A/Chicken/Hong Kong/715.5/2001 (H5N1) (H5N1/01) was isolate from chicken in 2001 in Hong Kong, the NCBI taxon ID was 196434 (Bi et al., 2011; Dong et al., 2011; Teng et al., 2016).

To track the H9N2 viruses, the genetic origin of the subtype was characterized based on phylogenetic analysis of eight segments (Figure 1), which was constructed using the maximum Likelihood method in MEGA6.0 (Tamura et al., 2013) with 1, 000 bootstrap replicates. The reference sequences used for genetic comparison were obtained from GenBank. Based on the phylogenetic analysis, three viruses (Gs/14, Ck/15, and Mi/14) belonged to genotype B69, which is a predominant genotype circulating in China and showed more efficient replication in chickens, isolate Sw/08 belonged to genotype B35 and isolate Hu/04 fell into genotype G9 (Figure 2) (Dong et al., 2011; Pu et al., 2015; Teng et al., 2016).

Amino acid mutation analysis showed that all viruses had 226G and 228R in the HA protein at the receptor binding region, and no deletion in the NA stalk regions was observed in these five H9N2 viruses. However, Gs/14, Ck/15, and Mi/14 shared similar amino acid sequences as compared to Sw/08 and Hu/04 (Table 1). In detail, PB2 (598V, 676M), PA (356R), NP (377N, 406V), NA (80N, 125S, 184Y, 199T, 356D), M1 (37A, 95K, 140A, 142G, 224N, 242N, 247L), M2 (16E, 21G, 31N, 68M, 82N), NS1 (47S, 80S, 103L), and NEP (14T, 21R, 49A) were shown in Table 1. In addition, mammalian adaptation associated mutations were observed in the PB2 gene of Mi/14 (A588V and D701N) and Sw/08 (D701N) (Sediri et al., 2015; Xiao et al., 2016) viruses. R340K was observed in the PB2 of Mi/14 and Sw/08 viruses, 522V was observed in PB1 of Mi/14 virus (Neumann et al., 2014), which is a potential virulence-related site. PA K356R which increases replication and pathogenicity, was observed in Gs/14, Ck/15, and Mi/14 (Xu et al., 2016).

**Table 1:** Amino acid mutations in the five characterized H9N2 viruses.

Segment	aa position	Amino acid substitution				
		Sw/08	Hu/04	Gs/14	Ck/15	Mi/14
PB2	87	D	D	N	D	D
	271	T	S	T	T	T
	340	K	R	R	R	K
	396	E	E	E	E	K
	588	A	A	A	A	V
	598	T	T	V	V	V
	627	E	E	E	E	E
	676	T	T	M	M	M
	701	N	D	D	D	N
PB1	197	K	K	R	R	K
	322	I	I	I	I	M
	434	T	T	N	N	T
	525	I	I	I	I	V
PB1-F2	51	M	T	T	T	T
	79	R	L	R	R	R
PA	70	A	A	V	A	A
	100	V	V	V	A	A
	356	K	K	R	R	R
	394	D	D	D	N	N
	594	S	S	R	S	S
	614	N	N	N	N	S
HA	191	N	N	N	N	H
	226	G	G	G	G	G
	228	R	R	R	R	R
NP	352	V	V	M	M	M
	377	S	S	N	N	N
	406	I	I	V	V	V
NA	80	K	N	N	N	N
	125	G	G	S	S	S
	184	H	H	Y	Y	Y
	199	K	K	T	T	T
	248	G	G	G	G	X
	356	S	N	D	D	D
M1	37	T	T	A	A	A
	95	R	R	K	K	K
	140	T	T	A	A	A
	142	V	V	G	G	G
	219	I	I	V	V	V
	224	S	S	N	N	N
	242	K	K	N	N	N
	247	M	M	L	L	L
M2	16	G	E	E	E	E
	21	D	G	G	G	G
	31	S	S	N	N	N
	68	V	V	M	M	M
	82	S	N	N	N	N
NS1	47	G	G	S	S	S
	80	T	T	S	S	S
	103	S	L	L	L	L
	106	L	I	I	L	V
	163	L	L	L	L	V
	212	P	P	S	S	s
	216	P	P	T	T	T
NEP	14	M	M	T	T	T
	21	G	G	R	R	R

### Pathogenicity of H9N2 Viruses in Chickens

**FIGURE 3:**
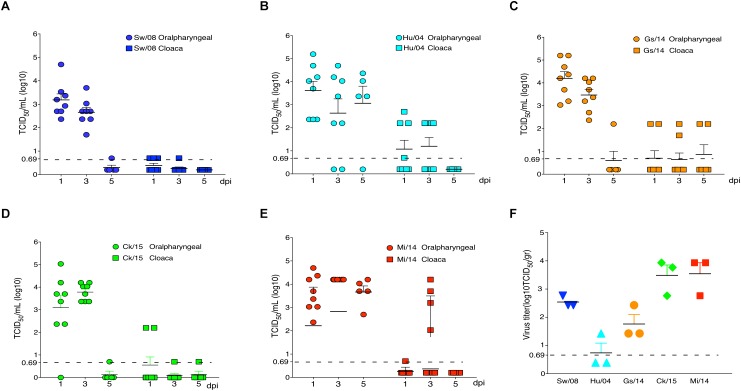
Replication of H9N2 viruses in chickens. Oropharyngeal and cloaca samples from the strains Sw/08 **(A)**, Hu/04 **(B)**, Gs/14 **(C)** and Ck/15 **(D)** and Mi/14 **(E)** viruses were tittered by TCID_50_. Virus load in the lung samples when infection at 3 dpi **(F)**. gr, grams.

All five H9N2 viruses replicated well in the oropharyngeal samples at 1 and 3 dpi; however, very low virus levels were detected from the oropharyngeal samples of Sw/08, Gs/14, and Ck/15 viruses at 5 dpi. The Hu/04, Gs/14, and Mi/14 viruses were excreted through the feces (Figure 3A–E). Meanwhile, the viral titers of lung samples at 3 dpi were also homogenized and titrated on MDCK cells, all five H9N2 viruses except Hu/04 replicated well in lungs. Compare to Sw/08 and Gs/14, Ck/15, and Mi/14 show a higher replication ability in chicken lungs (Figure 3F).

**FIGURE 4:**
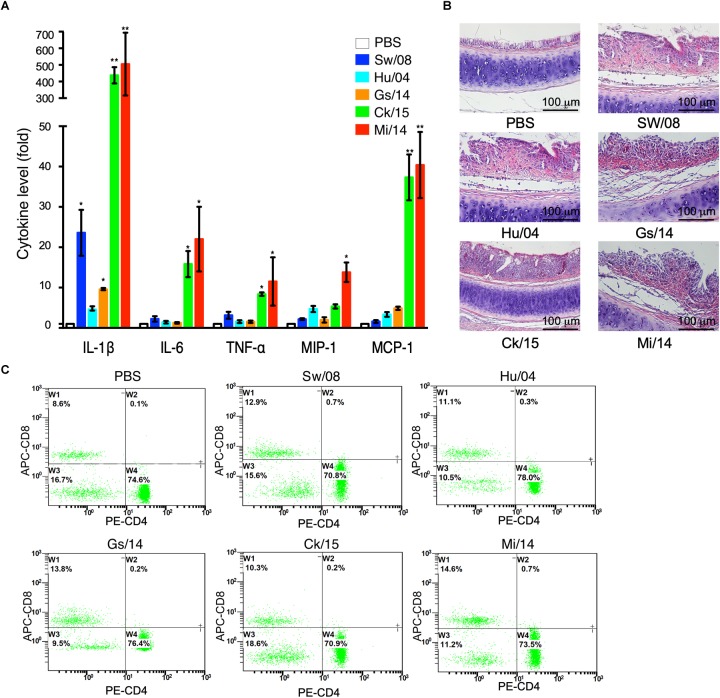
Pro-inflammatory responses and pathogenicity of the H9N2 viruses in chickens. **(A)** From the homogenized lung samples infected the five H9N2 viruses, the mRNA levels of IL-1β, IL-6, TNF-α, MIP-1, and MCP-1α were determined. **(B)** Histopathological examination with the infected tracheal samples by H&E staining. **(C)** FACS analysis to quantify CD_4_^+^ and CD_8_^+^ T cells. The data represent mean values ± SD from three independent experiments in triplicate. ^∗^*p* < 0.05, ^∗∗^*p* < 0.01.

To determine the pro-inflammatory response of the five viruses, the 3 dpi lung samples were homogenized to quantify the mRNA levels of IL-1β, IL-6, TNF-α, MIP-1, and MCP-1α (Figure 4A). In detail, the IL-1β mRNA level of Ck/15 and Mi/14 increased more than 400-fold compared to the PBS negative control and were also significantly higher than the other three H9N2 strains (*p* < 0.01). The mRNA levels of IL-6 (*p* < 0.05), TNF-α (*p* < 0.05), MIP-1 (*p* < 0.05), and MCP-1α (*p* < 0.01) also increased at least 10-fold. All H9N2 viruses caused severe tracheal lesions, with an increase in goblet cells and exudative inflammation, with a mean pathogenic score of 4 (Figure 4B). The mean amounts of CD_8_^+^ T cells were increased to 0.70% from 0.13% when chickens were infected with Sw/08 or Mi/14 (Figure 4C) (Table 2).

**Table 2:** FACS analysis to quantify CD_4_^+^ and CD_8_^+^ T cells.

	PBS	Sw/08	Hu/04	Gs/14	Ck/15	Mi/14
CD_4_^+^ T cells	79.63 ± 3.54	72.97 ± 1.93	75.10 ± 3.72	76.70 ± 0.61	72.90 ± 4.45	65.67 ± 8.70^∗^
CD_8_^+^ T cells	16.10 ± 6.70	14.40 ± 2.02	11.73 ± 1.83	11.23 ± 3.61	13.63 ± 5.10	19.27 ± 8.00
CD_4_^+^CD_8_^+^ T cells	0.13 ± 0.06	0.67 ± 0.06^∗^	0.23 ± 0.06	0.23 ± 0.06	0.23 ± 0.06	0.70 ± 0.10^∗^

*^∗^Represents significant changes compared to the amount of PBS control group (p < 0.05).*

### Pathogenicity of H9N2 Viruses in Mice

**FIGURE 5:**
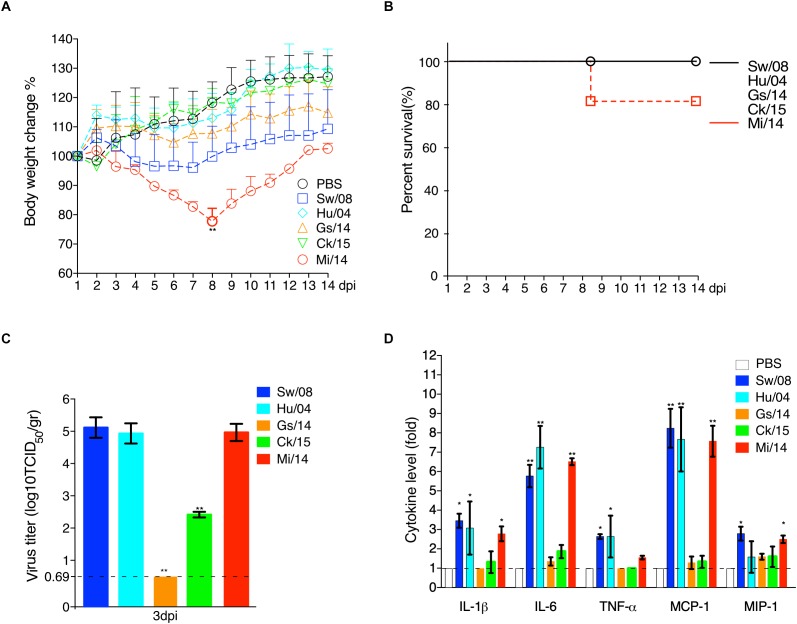
Pathogenicity of H9N2 viruses in mice. The body weight loss **(A)** and survival **(B)** were monitored daily until 14 days after inoculation. Virus load in the lung samples when infection at 3 dpi **(C)**. The pro-inflammatory cytokines levels of the mice lung samples at 3 dpi were analyzed by commercial sandwich ELISA kits **(D)**. The data are represented as mean values ± SD from three independent experiments in triplicate. ^∗^*p* < 0.05, ^∗∗^*p* < 0.01. gr, grams.

To evaluate the viral replication in lung samples, three mice from each group were euthanized at 3 dpi (Figure 5). Prior to necropsy, Mi/14 virus caused clinical signs of ruffled fur and depression. Remarkably, 20% mortality and significant body weight loss were obeserved at 8 dpi (*p* < 0.01). However, the other viruses caused no major clinical signs (Figure 5A). All the H9N2 viruses replicated in lungs without prior adaptation, except Gs/14. In detail, at 3 dpi, the lung of the mice infected with strain Sw/08 held a high virus load with a mean titer of 1.48 × 10^5^ TCID_50_/gr, strain Hu/04 with a mean titer of 6.01 × 10^4^ TCID_50_/gr, Ck/15 virus with a mean titer of 1.41 × 10^2^ TCID_50_/gr, Mi/14 virus with a mean titer of 8.60 × 10^4^ TCID_50_/gr. The Sw/08 and Mi/14 viruses showed a higher replication in the lungs of mice, which was 10 to 1, 000-fold higher, as compared with Hu/04 and Ck/15 (*p* < 0.01) (Figure 5C), which represented B35 (isolate Sw/08), B69 (isolates Gs/14, Ck/15, and Mi/14) and G9 (isolate Hu/04) genotypes respectively.

Meanwhile, the mice lung samples at 3 dpi were used to check the level of cytokines. The results showed that the mammal-origin Sw/08, Hu/04, and Mi/14 induced stronger pro-inflammatory responses, as indicated by quantifying IL-1β, IL-6, TNF-α, MCP-1, and MIP-1α levels using ELISA, as compared with cytokine increases induced by Gs/14 and Ck/15 (Figure 5D).

## Discussion

Different isolates of H9N2 AIV from different genotypes (B69, G1, and B35) were chosen to study the relationship between genotypes and pathotypes. Since 2010, genotype B69 has been predominant in chickens and ducks (Teng et al., 2016) and B69 genotype have been shown to provide internal genes for the emerging H7N9 and H10N8 viruses in zoonotic infections (Gao et al., 2013; Chen et al., 2014b). High genetic compatibility was indicated in the B69 genotype, as compared to other IAVs.

H9N2 viruses infect many species (Group, 2013). In mice, the strains Mi/14 (genotype B69) and Sw/08 (genotype B35) showed a higher pathogenicity and a stronger pro-inflammatory response (Figure 5). Obviously, Mi/14 infection showed a remarkable weight loss at 8 dpi. Moreover, 20% of mice died at 8 dpi. This suggested that the Mi/14 virus was more virulent in mice. In chickens, the isolates Mi/14 (genotype B69) and Ck/15 (genotype B69) showed a higher pathogenicity and a stronger pro-inflammatory response (Figure 3, 4). Interestingly, the higher viral load in lungs showed a higher pro-inflammatory response. It was likely antigenic load to increase the inflammatory response to virus infection. Hu/08 showed a higher replication in mice but lower in chickens. The human-virus replicated at lower levels in chickens, it was probably that adaptation to humans will decrease the virus replication in chickens. Moreover, Gs/14 cannot replicate in mice lung but replicate well in chicken. Thus, these data indicate the same virus in different hosts showed a complicated pathotype pattern. It suggested that the pathogenicity of H9N2 viruses is strain-dependent.

Our data show that Sw/08, Hu/04, and Mi/14 replicate well in mice lungs, indicating that the mammal-origin viruses replicated at higher levels than the avian-viruses in mice (Figure 5C). Higher adaptation indicating it is an important step for the virus to transmit from human-to-human. Strikingly, the mammalian adaptation-associated mutation D701N was observed in the PB2 gene of Mi/14 and Sw/08 viruses. PA sequences of the Sw/08, Hu/04, Ck/15, and Mi/14 were 70A and 594S, while Gs/14 was 70V and 594S. In addition, R340K was first observed in the PB2 gene of Mi/14 and Sw/08 viruses. These could be a potential virulence determinants that require confirmation.

Extensive studies on how H9N2 viruses affect pre-inflammatory cytokines expression have also been performed (Geiss et al., 2001, 2002; Kash et al., 2004; Degen et al., 2006; Kobasa et al., 2007). Significant increases in IL-1β, IL-6, TNF-α, MCP-1, and MIP-1α in the lungs when H9N2 infected. However, the chicken results of decreased IL-1β induced by Hu/04 virus compare with Sw/08, Ck/15, and Mi/14. However, the Hu/04 virus in mice yielded the same level of IL-1β production compared to Mi/14 and Sw/08 viruses, indicating differences in host-specific responses to viral antigen. Taken together, these five cytokines may be key factors for pulmonary pathology. One reason for the differences in levels of IL-1β production is the ability for activation of the inflammasome complex upon recognition of viral antigen, such as PB1-F2 (McAuley et al., 2013). Interestingly, the inflammasome activation-associated mutation site 79R was observed in the PB1-F2 of all five H9N2 viruses except Hu/04 virus (Alymova et al., 2018) (Table 1). Our results indicated that even LPAI H9N2 viruses replicated well in mice and chickens, and could cause significant increases in pro-inflammatory cytokine expression (Figure 4A, 5D). Future reverse genetic assays to engineer point mutations may reveal molecular mechanism of the virulence of H9N2 viruses.

## Author Contributions

HC conceived and designed the study. HS, KW, and WY developed the methodology. QL, JY, QT, and XL acquired the data (provided animals, materials, regents, facilities, etc.). HS, KW, and HC analyzed and interpreted the data. HS and HC wrote, reviewed, and/or revised the manuscript. HC and ZL supervised the study.

## Conflict of Interest Statement

The authors declare that the research was conducted in the absence of any commercial or financial relationships that could be construed as a potential conflict of interest.
